# Hepaticogastrostomy as salvage treatment in a case of clinical failure of cholecystoduodenostomy due to tumoral obstruction of the cystic duct

**DOI:** 10.1055/a-2092-0393

**Published:** 2023-06-12

**Authors:** Sergi Quintana-Carbo, Maria Puigcerver-Mas, Daniel Luna-Rodriguez, Albert Garcia-Sumalla, Sandra Maisterra, Joan B. Gornals

**Affiliations:** 1 Endoscopy Unit, Department of Digestive Diseases, Hospital Universitari de Bellvitge, Barcelona, Catalonia, Spain; 2 Universitat de Barcelona, Catalonia, Spain; 3 Bellvitge Biomedical Research Institute (IDIBELL), Barcelona, Catalonia, Spain

A 75-year-old woman with a history of endometrial adenocarcinoma was admitted to the hospital with jaundice. A large retroperitoneal mass was diagnosed by imaging tests, and anatomopathological study confirmed a recurrence. An initial endoscopic ultrasound (EUS) was performed, showing a large mass infiltrating the duodenal wall and the pancreatic head, with both biliary and pancreatic ducts dilated. A transpapillary biliary drainage approach was unsuccessful due to tumor infiltration of the papilla.

A second attempt, 2 weeks later, via endoscopic retrograde cholangiopancreatography (ERCP) failed again, and it was decided to perform an EUS-guided transmural biliary drainage in the same session.

The option of a choledochoduodenostomy was not technically feasible due to the presence of tumor infiltration and vessel interposition at the optimal point of access. Thus, it was decided to perform EUS-guided gallbladder drainage using a lumen-apposing metal stent (LAMS; 10 × 10 mm Hot-AXIOS; Boston Scientific, Marlborough, Massachusetts, USA). A cholecystoduodenostomy was technically successful without incidence; however, no improvement in clinical signs or tests was achieved. LAMS patency was checked via direct cholecystoscopy using a pediatric gastroscope, with identification of the cystic duct ostium. An attempt at rendezvous, with guidewire advancement from the gallbladder toward the papilla was unsuccessful due to tumor obstruction of the cystic duct. A hepaticogastrostomy was therefore performed as a salvage biliary drainage technique. Finally, a partially covered metallic biliary stent, specific for hepaticogastrostomy (8 × 8 mm; Giobor Niti-S biliary covered stent; Taewoong Medical Co., Ltd, Gimpo, Gyeonggi, South Korea) was successfully placed ( Fig. 1 , Fig. 2 , Fig. 3 , Video 1 ).

**Fig. 1:**
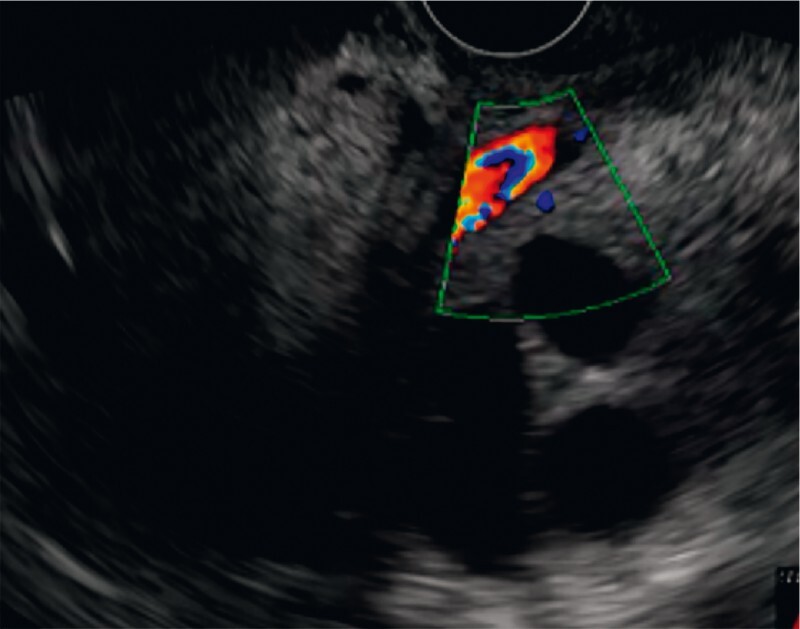
Endosonography image from the duodenal bulb showing tumoral infiltration and interposed vessels, excluding the possibility of performing a choledochoduodenostomy.

**Fig. 2:**
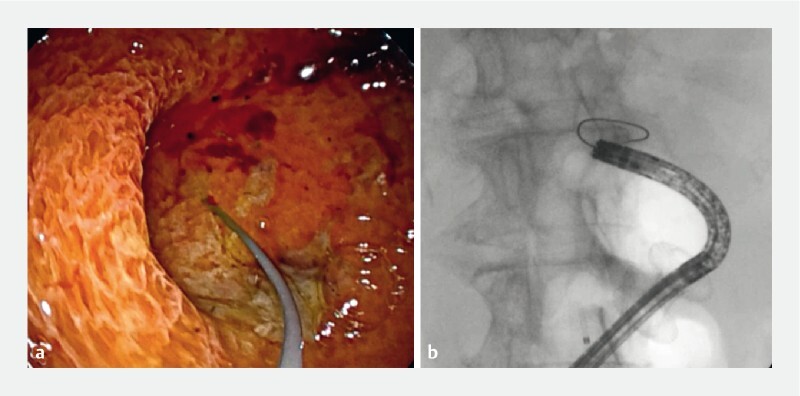
A rendezvous procedure was attempted from the gallbladder with the aim of antegrade advancement of a guidewire toward the papilla. **a** Endoscopy. **b** Fluoroscopy.

**Fig. 3:**
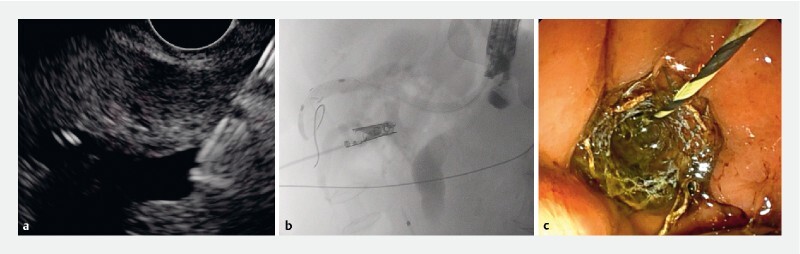
Hepaticogastrostomy, using a Giobor stent (Taewoong Medical Co., Ltd, Gimpo, Gyeonggi, South Korea). **a** Endosonography. **b** Fluoroscopy. **c** Endoscopy.

**Video 1** Hepaticogastrostomy as salvage treatment in a case of clinical failure of cholecystoduodenostomy due to tumoral obstruction of the cystic duct.


This is a case of a complex biliary drainage scenario due to malignant pathology that was not resolved via ERCP; the choledochoduodenostomy was not an option due to interposition of vessels and tumor, and EUS-guided gallbladder drainage could not offer any clinical improvement. It was not until successful hepaticogastrostomy had been achieved that the patient began to feel relief from her symptoms.

This case highlights the importance of considering all the different approaches of EUS-guided biliary intervention techniques in routine clinical practice, in order to increase the likelihood of clinical success 1 2 .

Endoscopy_UCTN_Code_TTT_1AS_2AD

## Notes

Endoscopy E-Videos

https://eref.thieme.de/e-videos
*E-Videos* is an open access online section of the journal *Endoscopy* , reporting on interesting cases and new techniques in gastroenterological endoscopy. All papers include a high-quality video and are published with a Creative Commons CC-BY license. Endoscopy E-Videos qualify for HINARI discounts and waivers and eligibility is automatically checked during the submission process. We grant 100% waivers to articles whose corresponding authors are based in Group A countries and 50% waivers to those who are based in Group B countries as classified by Research4Life (see: https://www.research4life.org/access/eligibility/ ). This section has its own submission website at https://mc.manuscriptcentral.com/e-videos
